# Editorial on the Special Issue Titled “Pathology and Diagnosis of Gynecologic Diseases”

**DOI:** 10.3390/diagnostics13223480

**Published:** 2023-11-20

**Authors:** Cinzia Giacometti, Kathrin Ludwig

**Affiliations:** 1 Pathology Unit, Department of Diagnostic Sciences, ULSS 6 Euganea, 35131 Padova, Italy; 2 Pathology Unit, Department of Medicine, University of Padova, 35128 Padova, Italy; kathrin.ludwig@aopd.veneto.it

## 1. Introduction

In the medical and diagnostic daily routine, gynecologic diseases present many different scenarios. Benign lesions may mimic malignant ones, and vice versa; hormonal effects (either menstrual, pregnancy, or therapy-related) may alter normal histology and create artifacts; systemic diseases, such as diabetes and connective tissue diseases, may influence the hormonal status and affect placentation and gestation; genetic imbalance (BRCA, *p53*, mismatch repair protein deficiency—dMMR) can cause breast, endometrial, and ovary cancer [1,2,3,4]. This female “cosmos,” in which so much is interconnected and happens due to something else, is complex, and diagnostic challenges, tricky differential diagnoses, and pitfalls are routinely encountered. For instance, in gynecologic pathology, the correlation between benign and malignant diseases in gynecologic pathology is well-described, with some overlap in ovarian endometrial cancer and endometriosis. Studies have shown that different malignant degeneration pathways can lead to the development of endometriosis-associated ovarian tumors of the endometrioid and clear cell histotypes [5,6]. In gynecologic pathology, benign diseases that can increase the risk of malignant disease and a variety of synchronous and multiple cancers are often encountered [7,8].

Researchers are still searching for new biomarkers to accurately predict common gynecologic tumors’ prognosis. For instance, there is a need for novel prognostic biomarkers to improve immunotherapy, such as ITGB2 in ovarian cancer. Despite significant advancements in immunotherapy, patients with epithelial ovarian cancer still respond poorly to it; this could be due to immunosuppression and the high heterogeneity of the disease. Therefore, more research needs to be conducted to understand the molecular mechanisms in the ovarian cancer tumor immune microenvironment and develop new therapies that can effectively heat the “cold” ovarian cancer and enhance the clinical efficacy of immunotherapy [9,10].

Female genital cancer can develop due to various factors, such as viruses, bacteria, and hormonal and genetic imbalances. In recent years, research has shown that the microbiome also plays a significant role in cancer development; additionally, HPV infection increases the risk of developing squamous cell carcinoma in the skin and mucous membranes [11,12,13,14].

Gynecologic pathology encompasses neoplastic diseases and pregnancy-related pathology, a peculiar field; this includes pre-implantation disease, which has gained significant importance due to the wider use of in vitro fertilization [IVF] techniques [15,16,17]. But also, during pregnancy, various diseases can endanger the health of both the fetus and the mother. Some examples include gestational diabetes, which can disrupt normal placental function [18,19,20], and extremely rare but aggressive diseases, such as complete hydatidiform mole [21,22].

## 2. An Overview of Published Articles

Bergamini et al. (Contributor 1) compare data from patients affected by endometriosis-associated ovarian tumors of the endometrioid and clear cell histotypes to investigate the hypothesis of a dichotomy in the histogenesis of these tumors. The study analyzed clinical data and tumor characteristics of 48 patients who were diagnosed with either pure, clear cell ovarian cancer or mixed endometrioid-clear cell ovarian cancer arising from endometriosis (or endometriosis-associated endometrioid ovarian cancer). The exact pathways that lead to the development of cancer from endometriosis are not yet fully understood. However, it is known that the development of ovarian cancer in women with endometriosis is a complex process that involves multiple steps. It begins with forming a precursor lesion, such as atypical endometriosis, which contains certain genetic and epigenetic mutations. Over time, these changes accumulate and are further compounded by the inflammatory, hyperestrogenic environment and oxidative stress in the endometriotic lesion, ultimately leading to the development of cancer. A particular subtype of endometriosis-associated ovarian cancer appears to develop slowly within an endometriotic cyst; this represents a subset of diseases where ultrasound could be useful in the early detection of malignant degeneration.

Sohn et al. (Contributor 2) analyzed tumors coexisting with endocervical polyps (ECPs) and studied the clinicopathological characteristics of ovarian and endometrial ECs involving ECPs. The study identified 429 ECPs, most associated with premalignant or malignant lesions in the uterine cervix, endometrium, and ovaries. No evidence of benign endometriosis, endometrial hyperplasia without atypia, or atypical hyperplasia/endometrial intraepithelial neoplasm within ECPs or the adjacent endocervical tissue was noted. According to the results, the involvement of ECPs by EC may have been due to an implantation metastasis from the ovarian or endometrial EC. The pathogenic mechanism of ECP involvement may have been implantation metastasis via transtubal and trans-endometrial cavity migration.

The article by Pongsuvareeyakul et al. (Contributor 3) reinforces the concept that determining the type and source of metastatic tumors is a crucial and potentially difficult task in pathology because it affects clinical decision-making and management of patients, as it may occur during an intraoperative exam when the site of origin of a clear cell tumor can pose an unpredictable diagnostic challenge. The authors presented a case of clear-cell mesothelioma, which originated in the uterine serosa and was initially misdiagnosed as clear-cell adenocarcinoma in the intraoperative frozen section. The tumor showed diffuse tubulocystic spaces of variable size lined by clear cells with moderate nuclear atypia. Immunohistochemical staining confirmed the diagnosis of clear-cell mesothelioma. This variant of epithelioid mesothelioma is an extremely rare neoplasm of the peritoneum and shares histomorphologic features overlapping with many other tumors, including carcinomas and non-epithelial neoplasms. Diagnosing peritoneal clear-cell mesothelioma is not always straightforward, despite known immunohistochemistry (IHC) markers. Due to its rarity, it may be easily confused with other clear-cell neoplasms, especially in intraoperative frozen sections. However, recognizing this rare entity is essential as the diagnosis could significantly affect the management considerations. The authors concluded that using an IHC panel judiciously can help distinguish this tumor from other mimickers.

Li, C, and colleagues (Contributor 4) conducted an integrated bioinformatic analysis to identify genes related to ovarian tumorigenesis and their immune characteristics in the ovarian cancer microenvironment. They filtered 332 differentially expressed genes from a database and identified 10 upregulated hub genes closely associated with ovarian tumorigenesis. The team proceeded to perform a survival and immune infiltration analysis that demonstrated that the upregulation of five candidate genes, ITGB2, VEGFA, CLDN4, OCLN, and SPP1, were correlated with unfavorable clinical outcomes and increased immune cell infiltration in ovarian cancer. Among these genes, ITGB2 correlated most with various immune cell infiltrations and strongly correlated with significant M2 macrophage infiltration while having a moderate correlation with CD4+/CD8+ T cells and B cells. This characteristic explains why ITGB2’s high expression was accompanied by immune activation but did not reverse carcinogenesis. Additionally, Western blotting and immunohistochemistry confirmed that ITGB2 was over-expressed in ovarian cancer tissues, primarily in the cytoplasm. In summary, ITGB2 may be a prognostic immunomarker for ovarian cancer patients.

The study by Kinoshita et al. (Contributor 5) explores the predominant histological subtype of breast mucinous carcinoma in older women, which is type B (hypercellular), while in younger women, it is type A (hypocellular). The characteristics of mucinous carcinomas of the same histological subtype may differ between older and younger women. The study aimed to systematically clarify mucinous carcinomas’ pathological and immunohistochemical features. Gross cystic disease fluid protein-15 (GCDFP-15) and eight other markers were used for immunostaining. The results showed that GCDFP-15 positivity was significantly higher in the older group compared to the younger group. Therefore, this study suggests that GCDFP-15 expression characterizes mucinous carcinomas in older women.

In the review by Trifanescu et al. (Contributor 6), the authors highlighted how the vagina harbors the highest number of bacteria, with a healthy profile dominated by Lactobacillus spp. On the other hand, the upper reproductive tract of females (consisting of the uterus, Fallopian tubes, and ovaries) has only a minimal number of bacteria. Although it was previously believed to be sterile, recent research has revealed the presence of a small microbiota in this region, with ongoing debates on whether it is a normal or pathological occurrence. It is noteworthy that the composition of the female reproductive tract’s microbiota is significantly influenced by estrogen levels. Increasingly, research suggests a correlation between the microbiome of the female reproductive tract and the development of gynecological cancers.

Kosmidis and colleagues (Contributor 7) discuss a series of cases of neoplasia in the anal and perianal region, highlighting the ongoing debate about whether young males and adult males should be vaccinated against HPV. Currently, there are no official guidelines regarding widespread vaccination for males or screening for anal SCC or HSIL (high-grade squamous intraepithelial lesion).

In their article, Ekemen et al. (Contributor 8) demonstrate the range of diagnostic tools available for predicting the outcome of IVF with the help of digital pathology. The authors explained how, in unexplained infertility and recurrent IVF failure cases, plasmacellular chronic endometritis and CD56 elevation (an increase in uterine NK cells) can be detected through three immunohistochemical stains; this helps in providing a specific treatment. This study also found that BCL-6 correlated well with CD56 positivity, even better than CD56 immunopositivity alone. Additionally, as BCL-6 positivity is associated with pelvic endometriosis, immunostaining of curettage material can allow for an easy diagnosis and protect individuals from more invasive interventions. However, further studies are required to evaluate BCL-6’s positivity in the endometrium.

Giacometti et al.’s (Contributor 9) research investigated the hypothesis that the absence or low expression of hENT1 in endothelial cells of all GDMd placentas could indicate a potential role in microvascular adaptive mechanisms. Due to the complex nature of the placental microenvironment, various pathways and metabolic mechanisms are likely to be affected by the alterations found at both cellular and phenotypic levels in GDM.

The article by Jung et al. (Contributor 10) reported an unusual case of placenta accreta, which was later determined to be an invasive hydatidiform mole. Unfortunately, it was not initially diagnosed as such. After radiologic examination, metastatic lung lesions were discovered, and the patient underwent six cycles of methotrexate administered at two-week intervals. The authors present this unexpected choriocarcinoma’s clinical and pathological characteristics with pulmonary metastasis, compare it to existing literature, and highlight the importance of thorough pathological examination.

## 3. Conclusions

The compilation of articles in this Special Issue on gynecologic pathology covers a wide range of research, reflecting the richness of this field. The studies adopted different methodologies, including observational approaches, such as case studies, molecular biology, and artificial intelligence. It is worth noting that the articles published in this Special Issue are from around the world, highlighting the relevance and importance of this publication. It offers readers a chance to discover research focused on extra-national contexts, which allows for a more complete understanding of the research field of gynecologic pathology.
